# Children’s job stability, intergenerational support, and the health of aging parents

**DOI:** 10.3389/fpubh.2025.1692730

**Published:** 2025-11-21

**Authors:** Yuqi Ye, Rui Chen

**Affiliations:** 1 School of Economics, Dongbei University of Finance and Economics, Dalian, Liaoning, China; 2 Institute for Northeast Full Revitalization, Dongbei University of Finance and Economics, Dalian, Liaoning, China

**Keywords:** children’s job stability, health of older population, gender difference, intergenerational support, healthy aging

## Abstract

**Background:**

The health of the older population is a core issue in China’s response to population aging. As the country with the largest older population worldwide, China faces mounting challenges in maintaining the physical and mental well-being of older adults, with families continuing to serve as the primary providers of care and support. Against this backdrop, examining how adult children’s job stability affects parental health is crucial for understanding intergenerational health transmission and shaping effective aging policies.

**Methods:**

Using data from the China Family Panel Studies (CFPS) from 2016 to 2022, this paper systematically examines the impact of children’s job stability for both physical and mental health of their parents, providing micro-level evidence on intergenerational transmission of health within families.

**Results:**

The study finds that children’s job stability significantly improves parents’ physical and mental health. Mechanism analysis shows that this effect primarily operates through enhanced financial support, emotional support, and perceived value support, which jointly contribute to better health outcomes for parents. Further analysis reveals that sons play a more pronounced role in improving parents’ physical health, while daughters have a stronger influence on mental health. This divergence stems mainly from the long-standing traditional gender-based divisions of caregiving roles, wherein sons tend to fulfill filial responsibilities through financial support, while daughters are more likely to provide emotional care. Heterogeneity analysis indicates that working hours influence intergenerational health transmission through the joint effects of the “income effect” and the “substitution effect.” Under conditions of overwork, the substitution effect tends to dominate, thereby weakening the positive health impact of children’s job stability on their parents.

**Discussion:**

This study has important implications for improving family support policies, addressing the challenges of an aging population, and promoting healthy aging. The findings suggest that enhancing children’s job stability not only strengthens their capacity to provide financial and emotional care, but also fosters a sense of intergenerational responsibility that benefits the well-being of aging parents. Policymakers should therefore prioritize labor market stability, promote work–life balance, and develop complementary social support mechanisms to reduce the overreliance on families, thereby creating a more sustainable framework for intergenerational health transmission.

## 1 Introduction

Population aging is one of the major social challenges of the 21st century. According to the World Health Organization, the global population aged 60 and above will double by 2050,^1^ placing unprecedented pressure on healthcare, care for older adults, and social security systems. As the country with the largest older population in the world, China is experiencing an accelerated aging process. By 2024, the number of people aged 60 and above had reached 310 million, accounting for 22% of the total population-twice the level in 2007.^2^ This number is now comparable to the entire population of the world’s third most populous country. The rapid aging trend has significantly increased health risks and long-term care demands among older adults, making the improvement of their health status and quality of life a central concern for both public policy and academic research.

Within the multilayered social security framework designed to mitigate the challenges of population aging, the family constitutes a foundational institution for safeguarding the health and welfare of older adults. The intrafamilial redistribution of resources and patterns of intergenerational support represent indispensable mechanisms in sustaining the well-being of family members (1). In China, where family-based elder care continues to dominate, approximately 90% of older adults rely primarily on home-based arrangements (2). Under this model, adult children serve as pivotal agents in the familial support system and in the transmission of intergenerational resources, assuming responsibilities that extend beyond material provision to encompass emotional reassurance and value affirmation.

Employment stability, in turn, is not merely indicative of sustained income, enhanced job security, and broader social protection, but also reflects individuals’ capacity to engage more actively in family relations and to provide consistent support (3). Consequently, the employment trajectories of adult children have implications that extend beyond their own economic accumulation and welfare entitlements: they shape both the ability and the willingness to fulfill filial responsibilities, thereby reinforcing parents’ health through financial contributions and psychosocial support (4). Nevertheless, the pathways through which adult children’s employment stability influences parental health remain underexplored, particularly in developing contexts where family-based elder care constitutes the predominant mode of old-age support. This lacuna highlights the necessity of a systematic analysis of the intrafamilial mechanisms linking employment stability and parental health outcomes.

From a theoretical perspective, children’s employment stability can influence parental health through multiple pathways. First, stable employment increases the continuity and predictability of income, enhancing children’s capacity to provide financial support, thereby alleviating parental stress related to living expenses and medical costs and improving physical health. In the context of traditional Chinese family norms, sons often bear greater economic responsibility and are regarded as the primary financial supporters; thus, their employment stability is expected to have a stronger effect on parental physical health. Second, stable employment is generally associated with lower job-related stress and better psychological well-being, enabling children to provide higher-quality emotional support and companionship. Daughters, with their typically stronger emotional expression and communication skills, often play a particularly significant and unique role in improving parents’ mental health (5, 6). Finally, employment stability not only reflects economic and emotional support but also represents a form of parental recognition of child-rearing success, contributing to parents’ perceived social value. This psychological satisfaction can enhance social engagement and promote healthier behaviors, thereby improving overall well-being.

Building on this background, this study employs data from the China Family Panel Studies (CFPS, 2016–2022) to examine how adult children’s employment stability affects the physical and mental health of parents aged 50 and above. It further explores the mechanisms of influence through economic, emotional, and perceived value support, while considering the differential impacts of children on the physical and mental health of their parents. Guided by this framework, this study addresses three key questions: (1) Does the employment stability of adult children significantly affect the physical and mental health of their parents? (2) Through which channels—economic, emotional, or perceived value support—does this effect operate? (3) Do sons and daughters exert differential effects on parents’ physical and mental health through distinct pathways? This study aims to elucidate how children’s employment stability affects their parents’ physical and mental health through the intergenerational transmission of family resources, to deepen the understanding of family-based support and health accumulation mechanisms, and to provide empirical evidence and policy implications for improving employment quality, strengthening family support systems, and promoting healthy aging.

This study makes three primary contributions. First, by examining the issue from the perspective of children’s employment stability, it extends the research frontier on intergenerational transmission of health and uncovers the role of employment stability within family support mechanisms, offering a new perspective and empirical evidence for the intersection of labor economics and health economics. Leveraging nationally representative CFPS data-which cover both urban and rural areas and households of diverse socioeconomic backgrounds-this study captures the realities of family support in China’s context of rapid aging and labor market transformation, thereby enhancing the external validity and policy relevance of the findings. Second, it identifies the spillover effects of employment stability on family members’ health, enriching the theoretical connotations and application scope of employment stability research, and confirming its applicability in a setting where the social security system is still evolving and families remain the main providers of care for older adults. Third, by incorporating gender differences and the moderating role of working hours, the study reveals differentiated roles of sons and daughters in supporting parental health and elucidates how labor intensity shapes these effects. Grounded in China’s unique cultural norms of gendered division of labor and its employment structure, the analysis offers targeted insights for optimizing family support structures and labor market policies in aging societies.

## 2 Literature review and hypothesis development

### 2.1 Literature review

In addition to stability of formal employment, recent literature in developing countries has increasingly examined the effects of flexible, informal or gig-type employment on family wellbeing and health. For instance, Liu et al. (7) find in China that while moderate levels of flexible employment can improve workers’ subjective well-being, excessive flexibility (and lack of protections) reduce it, with labor income acting as a key mediator. Similarly, a study of Chinese migrant workers demonstrates that self-employment—often a more flexible but less protected employment form—plays a complicated role in health inequality: those with lower education or income suffer more adverse health outcomes (8). In Sub-Saharan Africa, the emerging gig economy offers employment opportunities to women and youth through flexible work arrangements, but limited social security and unstable incomes raise concerns about long-term health and intergenerational support (9). These studies suggest that flexibility in employment, while offering economic opportunities, may come with trade-offs for health outcomes especially for older family members relying on consistent economic or emotional support.

Existing literature has conducted extensive and insightful investigations into the factors influencing the physical and mental health of older adults, covering a wide range of aspects such as individual income level, social security, health insurance, family structure, and intergenerational support. These studies can generally be categorized into three dimensions-economic, family, and societal perspectives.

First, in terms of economic capacity and social status, scholars emphasize the influence of individual economic capability and one’s position within the social structure on the health outcomes of older adults. An increase in personal income enables older adults to access greater material resources and invest more in their health, thereby improving quality of life and overall well-being (10). Conversely, widening income inequality tends to exacerbate health disparities, heighten feelings of relative deprivation, and increase psychological stress, which can negatively impact the health of the older population, particularly their mental health (11). Moreover, socioeconomic status serves as a critical measure of subjective well-being, self-worth, and social participation among older adults, providing substantial support for the improvement of both physical and mental health (12, 13).

Second, the impact of social institutions and the broader environment on older population health has also been widely examined. Research shows that social security systems-especially comprehensive pension schemes and healthcare coverage-offer essential institutional safeguards against health risks in old age, playing a key role in mitigating health vulnerabilities (14, 15). Beyond institutional security, numerous studies have investigated the effects of both community-based elder care and home-based elder care on the health of older adults (2, 16, 17). However, Zhang and Chen (18), in their analysis of the “New Rural Pension Scheme,” found that although public elder care can alleviate some of the pressures on family-based elder support, its impact remains limited, and family care continues to dominate in China. Consequently, exploring the determinants of older population health from a family perspective holds considerable practical significance.

Third, with the ongoing deepening of population aging, the family-as the fundamental social unit of health support for older adults-has gained increasing importance, especially regarding its internal support structures and intergenerational dynamics. Prior studies indicate that the effective allocation of family resources plays a pivotal role in safeguarding older adults physical and mental health (19). The extent of adult children’s involvement in providing material support and maintaining emotional ties directly affects their parents’ health and quality of life (20). Empirical evidence suggests that intergenerational support from children significantly improves parental physical health, mental well-being, and subjective happiness (21, 22). Regarding family structure, living arrangements are widely recognized as an important determinant of older population health (23). Older adults co-residing with their children tend to exhibit better health outcomes, whereas empty-nest elders and widowed individuals are generally more vulnerable (15, 24).

Additionally, gender differences in the provision of family support have attracted considerable attention. Recent studies have highlighted the evolving gender differentiation in intergenerational support. Sons generally provide greater economic assistance to their parents, while daughters more often enhance parents’ mental health through frequent interaction and emotional connection (5, 6). Longitudinal evidence from rural Anhui further shows that older parents cared for by daughters experience slower declines in daily living ability (ADL/IADL) than those cared for by sons (25). Research on migrant older populations also finds gendered differences in how emotional support networks shape older adults’ quality of life (26). Moreover, the influence of children’s gender on parental well-being appears stage-specific, becoming more evident after children’s marriage and childbirth, when daughters tend to bring greater happiness to parents—partly due to financial pressures related to “helping sons buy housing” (27). Overall, while sons remain more active in economic support, daughters’ contributions to emotional companionship and daily care have grown increasingly important, reflecting the gradual transformation of gender roles in contemporary Chinese families.

Building on existing research examining the impact of family support structures on the health of older adults, some studies have extended the analytical perspective to the labor market behaviors of family members, exploring their potential effects on the health status of other family members. Logically, as a key conduit for intergenerational resources, an individual’s performance in the labor market is closely tied to their capacity for intergenerational support. Strong labor market performance is often accompanied by better economic conditions, greater flexibility in time allocation, and more frequent emotional interactions within the family, thereby exerting a positive influence on the physical and mental health of family members.

However, the current literature predominantly focuses on a “top-down” influence pathway, with particular emphasis on how maternal employment affects the physical health and nutritional status of children (28, 29). In contrast, research on the “bottom-up” effects-how adult children’s labor market performance provides “reverse support” to parents and influences the health of older adults-remains relatively underdeveloped.

It is worth noting that employment stability, as a key indicator of labor market quality, not only concerns workers’ own development opportunities but also potentially shapes their ability to fulfill family responsibilities and allocate intergenerational resources. Existing studies have extensively explored the relationship between employment stability and individual health, including its impact on physical and mental health, disease incidence, and the long-term health consequences of early-career instability (30, 31). Nevertheless, literature that situates employment stability within the framework of intergenerational health transmission is relatively scarce.

In particular, against the backdrop of rising labor market uncertainty and a growing share of flexible employment, incorporating employment stability into the analytical framework of family intergenerational health transmission is of both theoretical and practical significance. Doing so helps clarify the micro-level mechanisms through which adult children’s employment stability affects parental health, deepens the understanding of family functions and aging-related challenges, and provides valuable empirical evidence and theoretical insights for optimizing employment stabilization policies and family support systems.

### 2.2 Hypothesis development

Building on the above literature review, this paper will analyze in detail the underlying mechanisms through which offspring’s employment stability influences parents’ physical and mental health, from the perspectives of economic support, emotional support, and perceived value support.

First, stable employment among adult children enhances the stability and sustainability of their income, thereby strengthening their economic capacity to support their parents. Economic support, as a core component of instrumental support, refers primarily to assistance provided by children to their parents in the form of cash, goods, or services, and constitutes an important channel for safeguarding the quality of life in later years (32). Specifically, higher and more stable earnings enable adult children to provide substantial and reliable financial support to their parents, alleviating economic burdens related to daily expenses and medical costs, thus creating favorable conditions for health improvement and reducing health risks arising from financial strain (19). Moreover, improved household economic conditions can enhance parents’ ability and willingness to participate in social and cultural activities-such as social gatherings and travel-which in turn bolster psychological well-being and foster social integration (33). Accordingly, economic support functions as a critical material safeguard for improving the physical and mental health of older adults: it mitigates health-related expenditures, enhances physical functioning, reduces life stress, and promotes psychological satisfaction. Relative to other forms of support, its marginal benefits-particularly in relation to physical health-are often greater due to its efficiency and alignment with the actual needs of health maintenance in old age (34). Furthermore, within the context of China’s traditional family structures and cultural norms, there is a pervasive son preference, whereby parents are more inclined to rely on sons for old-age support. From an economic perspective, in the long-standing socio-economic structure shaped by agricultural production and patriarchal norms, men are typically perceived as having greater household production capacity and bearing primary family economic responsibilities. Compared with women, they are often considered to yield higher marginal utility to the household (35). Consequently, sons are more likely to assume the role of the primary providers of intergenerational economic support to parents.

H_1_: Offspring’s employment stability enhances parents’ physical and mental health by strengthening economic support, with sons exerting a greater positive effect on parents’ physical health.

Second, non-instrumental support primarily refers to non-material assistance that children provide to their parents, such as emotional comfort, interpersonal interaction, and psychological care. It emphasizes the role of filial relationships, psychological support, and perceived value recognition in parent–child dynamics. Accordingly, this study explores the pathways through which offspring’s employment stability affects parents’ physical and mental health from the perspectives of emotional support and perceived value support. For adult children, employment stability implies lower work-related stress and reduced occupational uncertainty. A sound mental state and stable emotions foster greater family involvement, a stronger sense of responsibility, and more positive emotional engagement within the household (36). Conversely, employment instability may limit children’s autonomy over time allocation, reduce the frequency and quality of parental companionship, and thereby weaken emotional support. A relatively stable psychological state also facilitates the maintenance of harmonious parent–child relationships, enabling more effective communication and responsiveness to parents’ health needs, ultimately contributing to improvements in their physical and mental well-being. Furthermore, gender-based physiological differences and household role divisions mean that women often possess stronger emotional expression, communication skills, and empathy (37). As a result, daughters tend to assume greater responsibility for providing emotional support within families and are more adept at alleviating parents’ psychological stress through emotional interaction, thereby exerting a distinct and significant effect on improving parents’ mental health (38). Based on this reasoning, this study proposes the following hypothesis:

H_2_: Offspring’s employment stability enhances parental physical and mental health by strengthening emotional support, with daughters exerting a greater positive effect on parents’ mental health.

Third, perceived value support, as another dimension of non-instrumental support, reflects an individual’s sense of identity with their social role and psychological satisfaction. Within the family context, children’s employment stability not only signifies their economic capacity and emotional support but also concretely represents the outcomes of parental upbringing. This psychological satisfaction and sense of achievement enhance parents’ perceived value. Increased perceived value contributes to promoting parents’ social participation and health behaviors, thereby exerting a positive effect on their physical and mental health (12). Although sons and daughters exhibit notable gender-based differences in economic and emotional support, this study hypothesizes that there may be no significant difference between them regarding the impact of perceived value support on parents’ physical and mental health. This is primarily because perceived value support reflects parents’ recognition of their nurturing role and family achievements, which is not significantly related to specific gender role functions. Regardless of the child’s gender, employment stability serves as an important indicator of parental success. Moreover, with the gradual weakening of traditional “son preference” in modern society, parents’ perceived value of their children has become more balanced, resulting in no significant gender differences in influencing parents’ physical and mental health. Therefore, this study proposes the following hypothesis:

H_3_: Children’s employment stability enhances parents’ physical and mental health by strengthening perceived value support, and this mechanism does not significantly differ between sons and daughters.

## 3 Data and samples

The data used in this paper are drawn from the China Family Panel Studies (CFPS)^3^ for the years 2016–2022. There are three main reasons for selecting this dataset. First, the CFPS is a nationally representative, large-scale, and comprehensive longitudinal social survey that covers 29 provinces across China. It systematically captures both individual characteristics and household-level information, offering a clear advantage over other micro-level datasets in describing family structure and intergenerational dynamics. Second, the CFPS provides a well-constructed household information system that allows for accurate matching of parents and children, and includes key variables such as children’s labor market participation and parents’ health status-offering robust support for analyzing the link between employment stability and family health outcomes. Third, beginning in 2016, the CFPS began collecting data specifically related to intergenerational relationships-particularly interactions between parents and children-thereby providing critical empirical support for identifying the mechanisms through which offspring’s employment stability affects parental health.

This study utilizes data from the individual questionnaires and family relationship modules of the CFPS across multiple survey waves to extract key variables related to individual health status, employment stability, and intergenerational relationships. Each child is treated as the unit of observation, and their information is matched with that of their parents to construct a child–parent dyadic dataset. In addition, household-level economic information is incorporated from the CFPS economic status modules, enabling the construction of a comprehensive analytical sample that integrates individual, household, and intergenerational dimensions.

Based on this dataset, and following the research design of Wen and Huang (39), a subsample was further refined according to the following criteria: (1) the child must be at least 18 years old, and the parent must be at least 50 years old; (2) only children engaged in non-agricultural wage employment were retained, excluding those who are unemployed, enrolled in school, or not participating in the labor market, to ensure the accurate measurement of employment stability; (3) observations with missing values in key variables were excluded, and continuous variables were winsorized at the 1% level in both tails to mitigate the influence of outliers; and (4) the CFPS sampling weights were used to adjust the pooled samples across survey years, ensuring both representativeness and cross-year comparability. After applying these restrictions, the final analytical sample consists of an unbalanced pooled cross-sectional dataset comprising 31,210 observations.

The key explanatory variable in this study is offspring’s employment stability, proxied by a binary indicator of whether the child has signed a formal labor contract (1 = yes; 0 = no). The outcome variable is parental health, which is assessed from two dimensions: physical health and mental health.

Physical health is measured using a binary variable indicating whether the parent was hospitalized due to illness in the past 12 months (1 = yes; 0 = no). This measure captures longer-term health conditions and is less susceptible to short-term fluctuations caused by temporary illnesses such as colds or minor discomforts. Compared to indicators of short-term health, it provides a more stable reflection of overall physical well-being. In addition, the study incorporates alternative health indicators-including short-term physical health and self-rated health-for robustness checks.

Mental health is evaluated using the Center for Epidemiologic Studies Depression Scale (CES-D), which is based on self-reported frequency of various emotional and behavioral experiences over the past week. Respondents select from the following options: “Rarely or none of the time (<1 day),” “Some or a little of the time (1–2 days),” “Occasionally or a moderate amount of time (3–4 days),” and “Most or all of the time (5–7 days),” coded from 1 to 4, respectively.

It is important to note that the design of the CES-D scale in CFPS varies across survey waves. Specifically, CFPS 2016 employs the full 20-item version of the CES-D, while CFPS 2018, 2020, and 2022 adopt a shortened version consisting of 8 core items. To ensure comparability across survey years, this study follows the standard approach in the literature by reverse coding positively worded items (e.g., “I felt happy,” “I enjoyed life”), summing the scores, and then standardizing the aggregate score. A higher value of this standardized mental health variable indicates greater depressive symptoms, thereby reflecting a lower level of psychological well-being (40).

The selection of control variables in this study is structured around three key dimensions:

First, individual characteristics. To account for potential confounding factors at the individual level that may influence both offspring’s employment stability and parental health outcomes, a comprehensive set of control variables is included in the regression models. At the offspring level, controls include personal income, marital status, and educational attainment. At the parental level, controls encompass age, lifestyle habits (smoking and drinking), educational attainment, coverage by pension and medical insurance, urban–rural residency status, and individual income. These variables help isolate the effect of employment stability from other personal attributes that may also affect health outcomes.

Second, household characteristics. Given that this study focuses on the intergenerational relationship between offspring’s employment stability and parental health, household-level economic resources may serve as an important channel through which such effects operate. Accordingly, three dimensions of household economic status are controlled for: (1) per capita household income, capturing overall income level; (2) household net assets, reflecting accumulated wealth and economic foundation; and (3) household size, which accounts for differences in intergenerational support capacity and potential resource dilution effects due to the number of family members.

Third, regional characteristics. The level of regional economic development affects both employment opportunities and the strength of employment protection systems, while also shaping the provision of public health services. Additionally, the distribution of healthcare resources is a critical external factor influencing individual health outcomes. The development of medical infrastructure directly affects residents’ access to and utilization of healthcare services. To control for regional disparities, this study includes per capita GDP and the number of hospital beds per 10,000 residents at the regional level as proxies for economic development and healthcare capacity, respectively.

Detailed definitions and descriptive statistics of all variables are presented in Table 1.

**Table 1:** Summary statistics of main variables.

Variables	Variable definitions	*N*	Mean	Std. dev.	Min	Max
*old_health*	=1 if parents were hospitalized due to illness; 0 otherwise	31,210	0.343	0.475	0	1
*old_stdcesd*	Parental mental health	31,210	0	1	−1.706	6.600
*ch_stability*	=1 if offspring employment is stable; 0 if unstable	31,210	0.621	0.485	0	1
*ch_gender*	=1 if son; 0 if daughter	31,209	0.689	0.463	0	1
Individual characteristics						
*old_age*	Age of the parent	31,210	60.832	9.783	50	96
*old_manner*	=1 if having smoking or drinking habits; 0 otherwise	31,210	0.426	0.494	0	1
*old_edu*	=1 if illiterate/semi-illiterate; 2 if primary school; 3 if junior high school; 4 if senior high school/secondary technical school/vocational school; 5 if junior college; 6 if bachelor’s degree; 7 if master’s degree; 8 if doctoral degree	30,932	2.256	1.146	1	8
*old_insurance1*	=1 if has pension insurance; 0 if no insurance	31,210	0.611	0.488	0	1
*old_insurance2*	=1 if has health insurance; 0 if no insurance	27,645	0.940	0.238	0	1
*old_urban*	=1 if urban; 0 if rural	30,595	0.476	0.499	0	1
*old_income*	Personal income (in RMB, log-transformed)	31,210	4.341	4.477	0	12.976
*old_marry*	=1 if unmarried; 2 if married; 3 if cohabiting; 4 if divorced; 5 if widowed	29,491	2.285	0.863	1	5
*ch_income*	Personal income (in RMB, log-transformed)	28,398	10.484	0.942	0.693	14.407
*ch_edu*	Education level	30,782	9.744	1.430	1	8
*ch_marry*	=1 if unmarried; 2 if married; 3 if cohabiting; 4 if divorced; 5 if widowed	28,236	1.735	0.691	1	5
Household characteristics						
*lnfam_income*	Per capita household income (in RMB, log-transformed)	30,160	10.045	0.924	5.017	15.549
lnfam_asset	Household net wealth (in RMB, log-transformed)	28,432	12.578	1.699	0	17.939
*fam_child*	Number of children in the household	31,210	2.127	1.045	1	10
Regional characteristics						
*lnpro_gdp*	Per capita GDP (in RMB, log-transformed)	31,210	10.961	0.412	10.227	12.156
*lnpro_beds*	Hospital bed availability (per 10,000 people, log-transformed)	31,210	3.508	0.530	1.533	4.334

## 4 Method

In the baseline regression analysis, this study constructs the following two-way fixed effects model to examine the impact of offspring’s employment stability on their parents’ physical and mental health:


(1) $$\mathrm{old}\_\text{healt}h_{\mathrm{it}}=\alpha_{0}+\alpha_{1}\mathrm{ch}\_\text{stabilit}y_{\mathrm{it}}+\alpha_{2}X_{\mathrm{it}}+\delta_{i}+\delta_{t}+\varepsilon_{\mathrm{it}}$$

(2) $$\mathrm{old}\_\mathrm{ces}d_{\mathrm{it}}=\alpha_{0}+\alpha_{1}\mathrm{ch}\_\text{stabilit}y_{\mathrm{it}}+\alpha_{2}X_{\mathrm{it}}+\delta_{i}+\delta_{t}+\varepsilon_{\mathrm{it}}$$

As part of the extended analysis, this study examines whether the impact of offspring’s employment stability on parental physical and mental health varies by gender. To test for such heterogeneity, the following empirical model is employed:


(3) $$\begin{array}{c} \begin{array}{l} \mathrm{old}\_\text{healt}h_{\mathrm{it}}=\beta_{0}+\beta_{1}\mathrm{ch}\_\text{stabilit}y_{\mathrm{it}}+\beta_{2}\mathrm{ch}\_\text{stabilit}y_{\mathrm{it}}\times \mathrm{ch}\_\text{gende}r_{\mathrm{it}}\\ +\beta_{3}\mathrm{ch}\_\text{gende}r_{\mathrm{it}}+\beta_{4}X_{\mathrm{it}}+\delta_{i}+\delta_{t}+\varepsilon_{\mathrm{it}} \end{array} \end{array}$$

(4) $$\begin{array}{c} \begin{array}{l} \mathrm{old}\_\mathrm{ces}d_{\mathrm{it}}=\beta_{0}+\beta_{1}\mathrm{ch}\_\text{stabilit}y_{\mathrm{it}}+\beta_{2}\mathrm{ch}\_\text{stabilit}y_{\mathrm{it}}\times \mathrm{ch}\_\text{gende}r_{\mathrm{it}}\\ +\beta_{3}\mathrm{ch}\_\text{gende}r_{\mathrm{it}}+\beta_{4}X_{\mathrm{it}}+\delta_{i}+\delta_{t}+\varepsilon_{\mathrm{it}} \end{array} \end{array}$$

Where $\mathrm{old}\_\text{healt}h_{\mathrm{it}}$ denotes the physical health status of parent *i* in year *t*, and $\mathrm{old}\_\mathrm{ces}d_{\mathrm{it}}$ represents their mental health status, the key explanatory variable $ch\_\text{stabilit}y_{\mathrm{it}}$ captures the employment stability of child *i* in year *t*. $ch\_\text{gende}r_{\mathrm{it}}$ indicates the child’s gender, with females as the reference group. $X_{\mathrm{it}}$ is a vector of control variables. $\delta_{i}$ and $\delta_{t}$ represent individual and year fixed effects, respectively, controlling for unobserved individual heterogeneity and common time shocks. $\varepsilon_{\mathrm{it}}$ is the error term. Given the household-level structure of the dataset, standard errors are clustered at the household level across all panel regressions to address potential heteroskedasticity and serial correlation.

In the baseline model, a significantly negative *α*₁ suggests that children’s employment stability is associated with a lower probability of parental hospitalization and reduced CES-D scores-indicating that more stable employment among offspring improves both the physical and mental health of their parents.

In the heterogeneity analysis, *β*₁ captures the effect of daughters’ employment stability on parental health, while *β*₂ reflects the additional impact of sons relative to daughters. Thus, *β*₁ + *β*₂ represents the total effect of sons’ employment stability on parental physical and mental health.

## 5 Results

### 5.1 Empirical results

Columns (1)–(3) of Table 2 report the stepwise baseline regression results based on Equation 1. Specifically, Column (1) includes only individual and year fixed effects, while Columns (2) and (3) sequentially introduce personal and non-personal characteristics of both children and parents. The regression results show that, regardless of the control variable set used, offspring employment stability significantly reduces the probability of parental hospitalization, indicating a stable and positive effect on physical health.

**Table 2:** Empirical results.

Variables	*old_health*			*old_cesd*		
	(1)	(2)	(3)	(4)	(5)	(6)
*ch_stability*	−0.222***	−0.238***	−0.249***	−0.023	−0.061***	−0.063***
	(0.010)	(0.012)	(0.013)	(0.019)	(0.021)	(0.023)
Indiv. char.	No	Yes	Yes	No	Yes	Yes
Other char.	No	No	Yes	No	No	Yes
Individual FE	Yes	Yes	Yes	Yes	Yes	Yes
Year FE	Yes	Yes	Yes	Yes	Yes	Yes
*N*	31,210	22,694	21,052	31,210	22,694	21,052
Adj. *R*^2^	0.442	0.449	0.456	0.442	0.462	0.466

Standard errors clustered at the household level are reported in parentheses. Individual characteristics include personal income, marital status, educational attainment, age, lifestyle habits (smoking and drinking), coverage by pension and medical insurance, urban–rural residency status, and individual income. Other characteristics include household characteristics (per capita household income, household net assets, household size) and regional characteristics (per capita GDP and the number of hospital beds). The significance levels of 1%, 5%, and 10% are denoted by ***, **, and *, respectively. The results indicate that children’s employment stability has an improving effect on both the physical and mental health of their parents.

Columns (4)–(6) present the corresponding stepwise regression results based on Equation 2, which focuses on mental health outcomes. As additional controls related to children’s and parents’ characteristics are incorporated, offspring employment stability consistently shows a significant negative association with parents’ CES-D depression scores, suggesting a robust and positive effect on mental health.

In terms of economic significance, the estimates from Columns (3) and (6) provide meaningful insights. In Column (3), the coefficient for offspring employment stability is −0.249, indicating that, on average, parents whose children hold stable jobs are 24.9% less likely to be hospitalized compared to those whose children are in unstable employment. Given that the mean hospitalization rate in our sample is 34.3%, this suggests that parental hospitalization probability decreases from roughly 34% to about 9% when children have stable employment. This magnitude highlights the strong intergenerational health linkage within families, where children’s stable employment substantially improves parents’ health status. Similarly, the coefficient in Column (6) is −0.063, suggesting that a child’s transition from unstable to stable employment is associated with a 0.063 standard deviation decrease in the parent’s CES-D depression score. These results underscore the economically meaningful and beneficial role that offspring’s employment stability plays in improving both the physical and mental health of their parents.

### 5.2 Robustness analysis

#### 5.2.1 Alternative specifications of key variables

Columns (5) and (6) report the results based on these alternative definitions. The findings consistently show that offspring employment stability continues to exert a significant and positive effect on both the physical and mental health of their parents, confirming the robustness of the core results.

In the baseline regression, key variables for offspring employment stability and parental health are constructed based on the research design and prior literature. To address potential biases from alternative definitions, this study adopts different measures for both employment stability and health. The results are shown in Table 3.

**Table 3:** Alternative specifications of key variables.

Variables	*health1* (1)	*health2* (2)	*health3* (3)	*ave_cesd* (4)	*old_health* (5)	*old_cesd* (6)	*old_health* (7)	*old_cesd* (8)
Panel A. Replacing the dependent variable								
*ch_stability*	−0.153***	0.040***	−0.038***	−0.033**				
	(0.035)	(0.015)	(0.015)	(0.015)				
*N*	21,051	20,710	20,712	21,052				
Adj. *R*^2^	0.523	0.391	0.381	0.699				
Panel B. Replacing the independent variable								
*stability1*					−0.191***	−0.143***		
					(0.012)	(0.021)		
*N*					21,052	21,052		
Adj. *R*^2^					0.443	0.470		
Panel C. Replacing the independent variable								
*stability2*					−0.169***	−0.055***		
					(0.013)	(0.022)		
*N*					19,566	19,566		
Adj. *R*^2^					0.507	0.474		
Panel D. Replacing the independent variable								
*stability3*					−0.054***	−0.059***		
					(0.011)	(0.017)		
Panel E. Replacing the independent variable								
*Stability4*							−0.030***	−0.023***
							(0.007)	(0.011)
*N*	21,051	20,710	20,712	21,052	17,158	17,158	22,181	22,181
Adj. *R*^2^	0.523	0.391	0.381	0.699	0.330	0.526	0.406	0.495
Control	Yes	Yes	Yes	Yes	Yes	Yes	Yes	Yes
Individual FE	Yes	Yes	Yes	Yes	Yes	Yes	Yes	Yes
Year FE	Yes	Yes	Yes	Yes	Yes	Yes	Yes	Yes

The significance levels of 1%, 5%, and 10% are denoted by ***, **, and *, respectively. Standard errors clustered at the household level are reported in parentheses. After a series of robustness checks, the improving effects of children’s employment stability on parents’ physical and mental health remain significant, confirming the robustness of the results.

Panel A reports regressions with alternative dependent variables. Columns (1)–(3) use self-rated health (*health1*), perceived health change (*health2*), and recent physical discomfort (*health3*) as substitutes for physical health, while Column (4) employs the average CES-D score (*ave_cesd*) for mental health. Across all specifications, offspring employment stability consistently improves parents’ physical and mental health.

Panels B–E replace the key explanatory variable with alternative measures of employment stability. Panel B classifies labor dispatch or intermediary contracts as unstable; Panel C further treats weekly working hours below 35 as unstable; Panel D uses job changes as a proxy, coding zero changes as stable. Columns (5) and (6) show that the results remain robust under these alternative definitions, confirming the positive effect of offspring employment stability on parental health. Panel E develops a multidimensional employment stability index that captures labor continuity, income stability, and other related aspects.^4^ This reduces measurement errors from single indicators and yields results consistent with the baseline in both sign and significance.

#### 5.2.2 Excluding potential confounding factors

Although the baseline regressions are based on preprocessed data, potential confounders may still bias the estimated effect of offspring employment stability on parental health. To address this, the study applies additional sample restrictions, excluding individuals with limitations in activities of daily living (ADL), older parents who are widowed/divorced and living alone without children, and observations potentially affected by major public health events. The corresponding results are shown in Table 4.

**Table 4:** Excluding potential confounding factors.

Variables	ADL < =3		Parents living alone		Parents with ADL		Severe health shock	
	*old_health* (1)	*old_cesd* (2)	*old_health* (3)	*old_cesd* (4)	*old_health* (5)	*old_cesd* (6)	*old_health* (7)	*old_cesd* (8)
*ch_stability*	−0.256***	−0.062***	−0.249***	−0.065***	−0.256***	−0.063***	−0.431***	−0.085*
	(0.014)	(0.023)	(0.014)	(0.023)	(0.015)	(0.023)	(0.025)	(0.050)
Control	Yes	Yes	Yes	Yes	Yes	Yes	Yes	Yes
Individual FE	No	No	Yes	Yes	Yes	Yes	Yes	Yes
Year FE	Yes	Yes	Yes	Yes	Yes	Yes	Yes	Yes
*N*	18,483	18,483	19,383	19,383	17,074	17,074	11,708	11,708
Adj. *R*^2^	0.442	0.440	0.455	0.450	0.444	0.435	0.509	0.485

The significance levels of 1%, 5%, and 10% are denoted by ***, **, and *, respectively. Standard errors clustered at the household level are reported in parentheses. After controlling for a series of potential confounding factors, children’s employment stability continues to exert a significant improving effect on parents’ physical and mental health, consistent with the baseline regression results.

Columns (1)–(2) exclude respondents with ADL difficulties, defined as having scores of 3 or below on seven daily activities (going out, eating, cooking, using transport, shopping, cleaning, doing laundry). Columns (3)–(4) exclude older parents living alone after widowhood or divorce, whose health is more affected by social isolation and living arrangements. Columns (5)–(6) exclude both groups simultaneously. Columns (7)–(8) further drop post-2020 observations to avoid bias from major public health shocks.

Across all specifications, results remain consistent: offspring employment stability continues to exert a significant improvement effect on both parental physical and mental health, confirming robustness against these potential biases.

#### 5.2.3 Clustering level adjustment and outlier control

To further account for potential bias arising from regional environmental factors, this study adjusts the clustering level of standard errors from the household level to the village/community level. Columns (1) and (2) of Table 5 present the corresponding regression results. After clustering at the village level, the effect of offspring employment stability remains statistically significant, continuing to reduce the probability of parental hospitalization and CES-D depression scores. This suggests that the positive health effects of employment stability are robust to changes in clustering level.

**Table 5:** Clustering level adjustment and outlier control.

Variables	Clustering level adjustment		*old_cesd*			
	*old_health* (1)	*old_cesd* (2)	uppertrim2.5% (3)	uppertrim5% (4)	upperwinsor2.5% (5)	upperwinsor5% (6)
*ch_stability*	−0.266***	−0.060**	−0.044***	−0.030**	−0.046**	−0.038**
	(0.015)	(0.025)	(0.017)	(0.015)	(0.020)	(0.018)
Control	Yes	Yes	Yes	Yes	Yes	Yes
Individual FE	Yes	Yes	Yes	Yes	Yes	Yes
Year FE	Yes	Yes	Yes	Yes	Yes	Yes
*N*	18,493	18,493	20,162	19,803	21,052	21,052
Adj. *R*^2^	0.460	0.464	0.463	0.495	0.477	0.496

The significance levels of 1%, 5%, and 10% are denoted by ***, **, and *, respectively. Standard errors clustered at the household level are reported in parentheses. After adjusting the clustering level and controlling for the influence of extreme values, the improving effect of children’s employment stability on parents’ mental health remains significant.

In the baseline regressions, the mental health variable is constructed by standardizing the total CES-D score. As shown in the descriptive statistics in Table 1, the standardized mental health variable ranges from −1.706 to 6.600 and exhibits noticeable right skewness. To address the potential influence of outliers, this study conducts robustness checks by applying both 2.5 and 5% right-side trimming and winsorization to the standardized CES-D variable. The results are reported in Columns (3) through (5) of Table 5.

The findings indicate that the positive association between offspring employment stability and parental mental health remains statistically significant after addressing extreme values, suggesting that the core conclusions are not driven by outlier observations and that the results exhibit strong robustness.

#### 5.2.4 Placebo test

**Figure 1:**
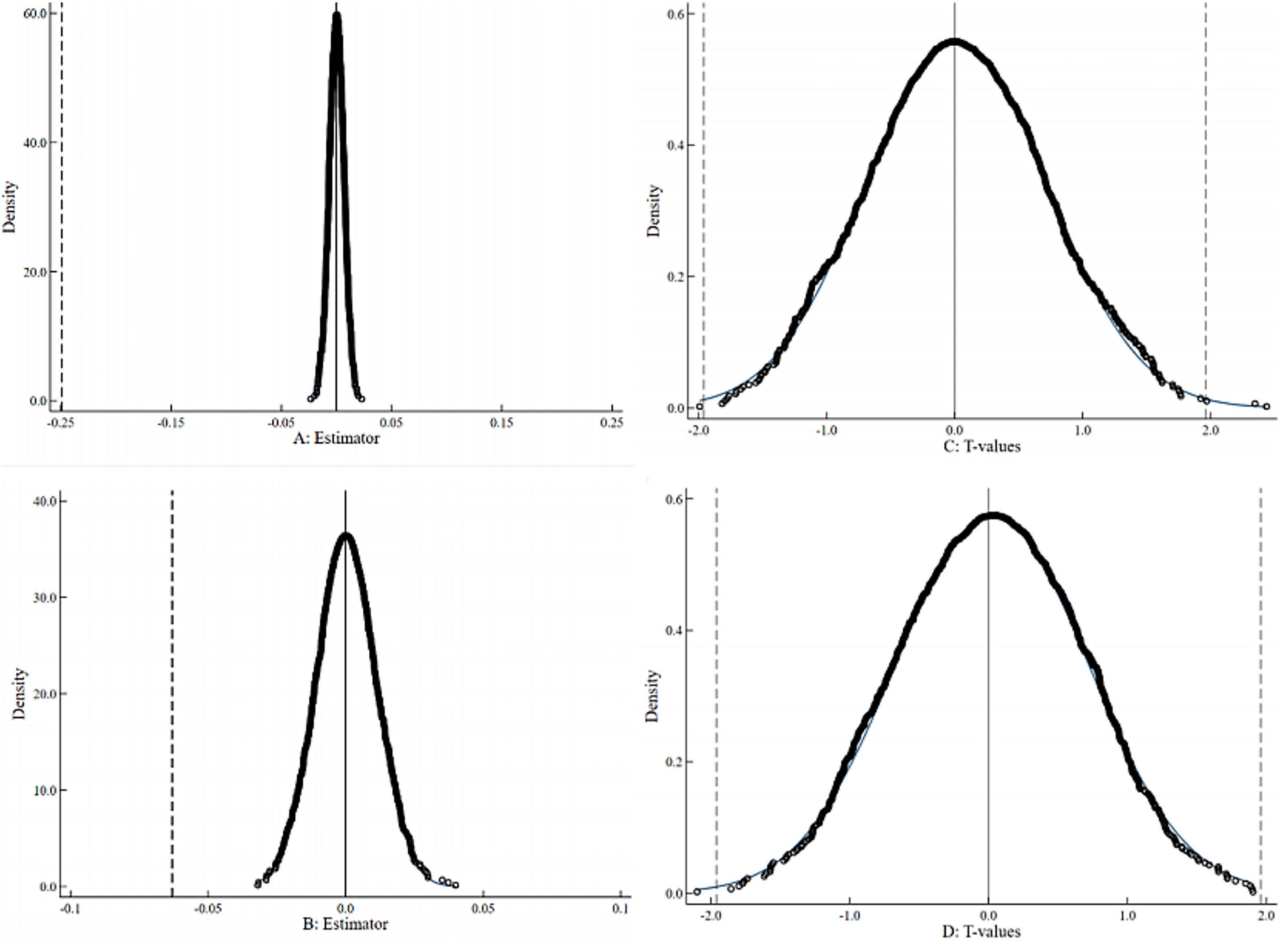
Placebo test. The figure is based on 1,000 random iterations with the random seed set to 123.

To further verify that the effects of offspring employment stability on parental health are robust and not driven by omitted variables, model misspecification, or random factors, this study conducts a placebo test following Li et al. (41). The employment stability variable is randomly reassigned across individuals while preserving its distribution, and regressions are re-estimated. If the placebo estimates are insignificant, the original findings are unlikely to result from chance. Figure 1 reports results from 1,000 replications: Panels A and C show the distributions of placebo coefficients for parental physical and mental health, while Panels B and D display the corresponding *t*-statistics. The placebo coefficients are normally distributed around zero, and the baseline estimates lie far outside these distributions. Most placebo *t*-values fall within [−2,2], indicating insignificance. Overall, the results clearly distinguish the true effects from random outcomes, confirming that the observed impacts on parental health are robust and credible.

#### 5.2.5 Endogeneity discussion

Although the preceding robustness checks strengthen the credibility of the regression results, the estimated effect of offspring employment stability on parental health may still be subject to endogeneity concerns arising from potential reverse causality or omitted variable bias. To address these issues and improve causal identification, this study adopts an instrumental variable (IV) approach to construct a more reliable identification strategy and mitigate potential endogeneity.

This study employs the establishment of China’s Free Trade Zones (FTZs) as an exogenous policy shock to construct an instrumental variable for identifying the causal impact of offspring employment stability. As a key strategic initiative to deepen reform and promote high-level opening-up, FTZs have expanded rapidly since the launch of the China (Shanghai) Pilot Free Trade Zone in 2013. To date, 22 FTZs have been established across eastern, central, and western regions of the country, forming a broad institutional innovation framework.

The FTZ policy generates substantial institutional dividends by promoting openness and innovation in investment, trade, and finance. First, the implementation of FTZs improves the overall business environment and standardizes labor practices, which in turn stabilizes business operations and enhances job security and career sustainability for workers. Second, the combined effects of institutional incentives and market mechanisms have accelerated industrial upgrading, improved job quality, and increased the alignment between workers’ skills and job requirements. These changes help mitigate involuntary job transitions and short-term unemployment driven by job insecurity and skill mismatches (42).

Moreover, FTZs exert significant spillover effects. Through improvements in factor allocation, institutional supply, and market expectations, FTZs positively influence surrounding regions by stimulating employment growth and structural optimization even outside the pilot zones (43). Therefore, the establishment of FTZs is a relevant instrument for employment stability, satisfying the requirement of instrument relevance.

On the other hand, as a region-specific policy aimed at driving institutional reform and high-quality economic development, the FTZ policy does not directly affect the physical or mental health of parents, thus satisfying the condition of exogeneity.

The corresponding instrumental variable regression model is specified as follows:


(5) $$\begin{array}{c} \begin{array}{l} \mathrm{old}\_\text{healt}h_{\mathrm{it}}=\mathrm{\lambda ch}\_\text{stabilit}y_{\mathrm{it}}+\Gamma X_{\mathrm{it}}+\delta_{i}+\delta_{t}+\varepsilon_{\mathrm{it}}\\ \mathrm{old}\_\mathrm{ces}d_{\mathrm{it}}=\mathrm{\lambda ch}\_\text{stabilit}y_{\mathrm{it}}+\Gamma X_{\mathrm{it}}+\delta_{i}+\delta_{t}+\varepsilon_{\mathrm{it}} \end{array} \end{array}$$

(6) $$\begin{array}{c} \mathrm{ch}\_\text{stabilit}y_{\mathrm{it}}=\mathrm{\gamma i}v_{\mathrm{it}}+\Gamma X_{\mathrm{it}}+\delta_{i}+\delta_{t}+\nu_{\mathrm{it}} \end{array}$$

Where Equation 5 represents the second-stage regression and Equation 6 the first-stage regression, the instrumental variable *iv_it_* is a multi-period DID indicator for the establishment of Free Trade Zones (FTZs). Specifically, *iv_it_* equals 1 if province *i* has implemented an FTZ policy in year *t* or any subsequent year, and 0 otherwise.

The two-stage regression results based on this instrumental variable are reported in Table 6, Panels A and B. Panel A presents the first-stage estimation, showing that the establishment of FTZs significantly increases offspring employment stability at the 1% significance level. Moreover, the first-stage *F*-statistic is 17.452, exceeding the critical value for weak instrument detection at the 10% level according to the Stock-Yogo test. Panel B reports the second-stage results, which indicate that, after addressing potential endogeneity, offspring employment stability continues to exert a significant and positive effect on both the physical and mental health of parents.

**Table 6:** Results of endogeneity discussion.

Variables	*ch_stability*	*old_health*	*old_cesd*	*old_health*	*old_cesd*	*old_health*	*old_cesd*
	(1)	(2)	(3)	(4)	(5)	(6)	(7)
Panel A. first-stage regression							
*iv*	0.091***						
	(0.022)						
Panel B. two-stage least squares (2SLS)							
*ch_stability*		−0.603***	−1.252***				
		(0.223)	(0.448)				
Panel C. parsimonious regression							
*iv*				−0.063***	−0.115***		
				(0.021)	(0.032)		
Panel D. semi-parsimonious regression							
*iv*						−0.032	−0.082
						(0.020)	(0.070)
*ch_stability*						−0.247***	−0.056**
						(0.013)	(0.023)
Control	Yes	Yes	Yes	Yes	Yes	Yes	Yes
Individual FE	Yes	Yes	Yes	Yes	Yes	Yes	Yes
Year FE	Yes	Yes	Yes	Yes	Yes	Yes	Yes
KP *F* value	17.452 {16.38}						
KP LM	34.378 [0.000]						
*N*	21,052	21,052	21,052	22,181	22,181	21,052	21,052

The significance levels of 1%, 5%, and 10% are denoted by ***, **, and *, respectively. Standard errors clustered at the household level are reported in parentheses. The weak identification test is conducted using the Kleibergen–Paap rk Wald *F* statistic, with the critical value at the 10% level provided by Stock and Yogo (2005) shown in parentheses. The underidentification test is based on the Kleibergen–Paap rk LM statistic, with the corresponding *p*-value reported in parentheses. The instrumental variable results indicate that, after addressing endogeneity, children’s employment stability continues to significantly improve parents’ physical and mental health.

This paper posits a correlation between the establishment of Free Trade Zones (FTZs) and employment stability of the subsequent generation. The first-stage regression results presented earlier have confirmed the correlation between the instrument and the endogenous explanatory variable. Building on this, following the methodology of Nunn and Qian (44), we further conduct a reduced-form regression to test the association between the instrument and the dependent variable. This approach provides preliminary evidence for the existence of a causal pathway and further strengthens the validation of the instrument’s relevance. The econometric model is specified in Equation 7, where the coefficient $\pi_{0}$ represents the reduced-form effect of the FTZ policy on parents’ physical and mental health. The corresponding regression results are reported in Table 6, Panel C. The results demonstrate a significant correlation between the instrument and the dependent variable, thereby further confirming the validity of the instrument.


(7) $$\begin{array}{c} \begin{array}{l} \mathrm{old}\_\text{healt}h_{\mathrm{it}}=\pi_{0}iv_{\mathrm{it}}+\pi_{1}X_{\mathrm{it}}+\delta_{i}+\delta_{t}+\varepsilon_{\mathrm{it}}\\ \mathrm{old}\_\mathrm{ces}d_{\mathrm{it}}=\pi_{0}iv_{\mathrm{it}}+\pi_{1}X_{\mathrm{it}}+\delta_{i}+\delta_{t}+\varepsilon_{\mathrm{it}} \end{array} \end{array}$$

This paper employs a partial reduced-form regression to test whether the instrument satisfies the exclusion restriction. Specifically, controlling for the endogenous variable-employment stability-the instrument is included as an explanatory variable in the baseline regression to examine whether it has a direct effect on parental health outcomes. If the instrument satisfies the exclusion restriction by affecting the dependent variable only indirectly through employment stability, its coefficient should be statistically insignificant, while the coefficient on the endogenous variable should remain significantly negative. The estimation equation for the partial reduced-form regression is specified in Equation 8, with results shown in Table 6, Panel D. The findings indicate that after including the instrument, the effect of offspring employment stability on parental hospitalization probability and depression scores remains significantly negative, whereas the instrument’s coefficients are not statistically significant. This suggests that the establishment of the Free Trade Zones does not directly impact parental health, satisfying the exclusion restriction and further supporting the instrument’s validity. Moreover, to further ensure robustness, we conducted a series of diagnostic tests on the instrumental variable. Specifically, the Kleibergen–Paap rk Wald F statistic, a robust first-stage *F*-statistic, was used to test for weak instruments. The result (*F* = 17.452) exceeds the 10% critical value (16.38) suggested by Stock and Yogo (2005), indicating that the instrument is not weak. Additionally, the Kleibergen–Paap rk LM statistic was employed to test for under-identification. The statistic is 34.378 with a *p*-value of 0.000, rejecting the null hypothesis of under-identification and confirming that the instrument provides sufficient explanatory power for the endogenous regressor.


(8) $$\begin{array}{c} \begin{array}{l} \mathrm{old}\_\text{healt}h_{\mathrm{it}}=\pi_{0}iv_{\mathrm{it}}+\pi_{1}\mathrm{ch}\_\text{stabilit}y_{\mathrm{it}}+\pi_{2}X_{\mathrm{it}}+\delta_{i}+\delta_{t}+\varepsilon_{\mathrm{it}}\\ \mathrm{old}\_\mathrm{ces}d_{\mathrm{it}}=\pi_{0}iv_{\mathrm{it}}+\pi_{1}\mathrm{ch}\_\text{stabilit}y_{\mathrm{it}}+\pi_{2}X_{\mathrm{it}}+\delta_{i}+\delta_{t}+\varepsilon_{\mathrm{it}} \end{array} \end{array}$$

Although the validity of the instrument has been verified through a series of tests above, considering that it is challenging to obtain instruments that fully satisfy the exogeneity assumption in practice, this paper further adopts the approach of Conley et al. (45) to relax the strict exogeneity requirement and employs Bayesian methods to assess the robustness of the estimates. Specifically, under the strict exogeneity assumption, the instrument *iv_it_* in Equation 8 does not directly affect the dependent variables $\mathrm{old}\_\text{healt}h_{\mathrm{it}}$ and $\mathrm{old}\_\mathrm{ces}d_{\mathrm{it}}$ , implying $\pi_{0}$ =0. However, in reality, the instrument may fail to fully meet the exclusion restriction. To address this, this study applies the Union of Confidence Intervals (UCI) and Local to Zero (LTZ) methods to evaluate whether the core regression results remain robust when the instrument and endogenous variable are strongly correlated but the instrument is not perfectly exogenous.

**Figure 2:**
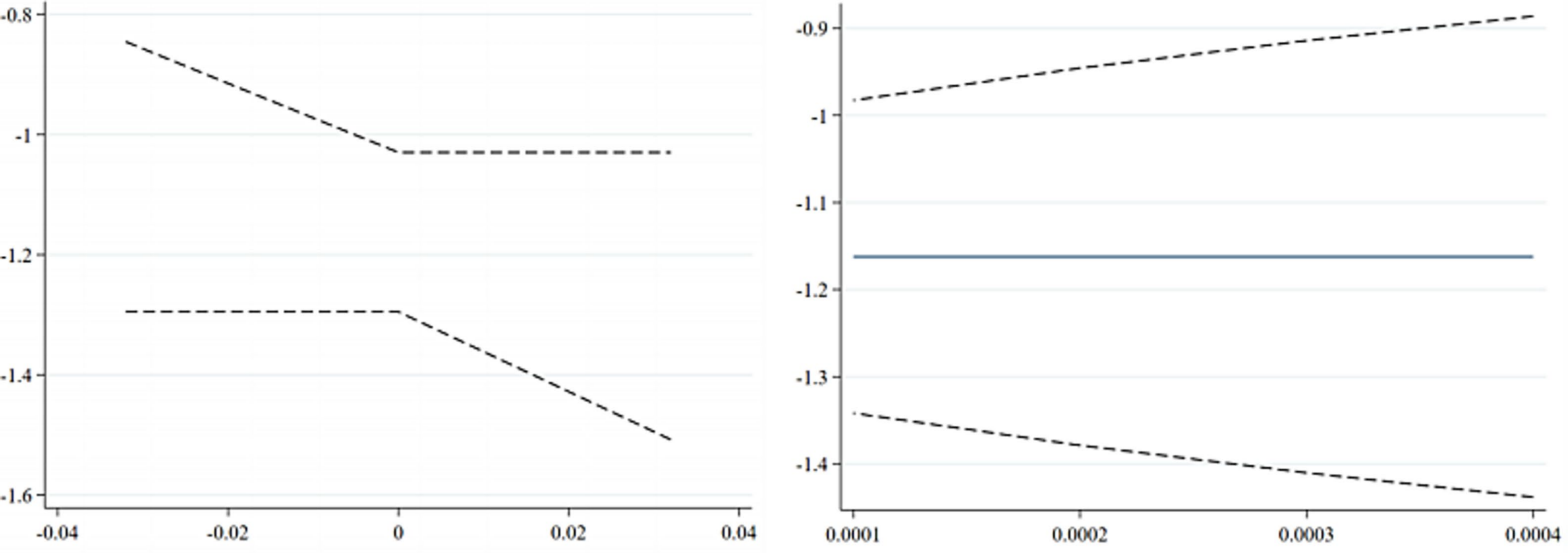
Estimated coefficients and confidence intervals from the UCL and LTZ methods.

Figure 2 presents coefficient estimates and confidence intervals derived from the UCI and LTZ methods. The left panel displays confidence intervals estimated via the UCI approach. Following the methodology of Ao et al. (46) and Xie et al. (47), the instrument is incorporated into the baseline regression to obtain estimates of its direct effect on the dependent variable, i.e., the plausible range of $\pi_{0}$ . As shown, even allowing for limited deviations from the exogeneity assumption, the 95% confidence intervals of the key explanatory variable’s estimates lie entirely in the negative region without crossing zero. This indicates that the estimates obtained using the Free Trade Zone policy as an instrument for employment stability remain robust under approximate exogeneity.

The right panel shows results from the LTZ method, which sets the standard error of $\pi_{0}$ as the variance of its distribution to characterize the degree to which the instrument may deviate from strict exogeneity. Under this framework, the estimated coefficient for the core explanatory variable remains significantly negative at the 95% confidence level. This further confirms that, even when relaxing the exclusion restriction, the identified effect of offspring employment stability on parental health remains statistically significant, underscoring the robustness of the causal inference.

## 6 Discussion

### 6.1 Mechanism analysis

The baseline regression results show that offspring employment stability has a significant improvement effect on both parents’ physical and mental health, making it an important factor in improving older population health outcomes. To further explore the underlying reasons and mechanisms, this paper examines the issue from the perspective of intergenerational family support. Based on the theoretical analysis and hypotheses discussed earlier, intergenerational support from children is divided into instrumental and non-instrumental dimensions, both crucial for parental health. Older population receiving more intergenerational support generally demonstrate better physical and mental health (32). Accordingly, this study systematically investigates how offspring employment stability influences family intergenerational support behaviors in terms of economic support, emotional support, and perceived value support.

It should be noted that in the CFPS questionnaire’s “Relationships with Children and Parents” module, respondents were asked identical questions separately about their children and parents, which may lead to inconsistencies in responses. Since the goal here is to identify the impact of offspring employment stability on family intergenerational support, the children’s responses are prioritized in data processing. If a variable is missing in the offspring sample but available in the parents’ sample, the parents’ responses are used to supplement. This approach maximizes sample retention while ensuring consistency in variable definitions and mechanism identification.

To systematically examine the role of economic support in the relationship between offspring employment stability and parental physical and mental health, this paper analyzes both the provision of economic support and the amount of economic support provided, aiming to comprehensively reveal how offspring employment stability affects intergenerational economic support within families. First, based on the questionnaire response to “In the past 6 months, have you provided financial assistance to your father/mother (child)?” a binary indicator (*support1*) reflecting whether economic support occurred is constructed, coded as 1 for “yes” and 0 otherwise. Second, using the response to “On average, how much money did you give your father/mother (child) each month in the past 6 months?” a continuous measure of economic support intensity (*support2*) is created. Regression results in Columns (1) and (2) of Table 7 show that employment stability significantly increases both the likelihood of children providing financial assistance to their parents and the amount of economic support given.

**Table 7:** Mechanism analysis.

Variables	Economic support		Emotional support			Perceived value support		
	*support1* (1)	*support2* (2)	*relation* (3)	*connect* (4)	*meet* (5)	*status* (6)	*satisfy* (7)	*happy* (8)
*ch_stability*	0.202***	0.395***	0.393***	0.308***	0.534***	0.078**	0.091***	0.524***
	(0.020)	(0.052)	(0.046)	(0.075)	(0.084)	(0.034)	(0.032)	(0.066)
Control	Yes	Yes	Yes	Yes	Yes	Yes	Yes	Yes
Individual FE	Yes	Yes	Yes	Yes	Yes	Yes	Yes	Yes
Year FE	Yes	Yes	Yes	Yes	Yes	Yes	Yes	Yes
*N*	21,034	15,932	20,631	21,052	21,011	21,052	21,052	21,052
Adj. *R*^2^	0.237	0.285	0.276	0.309	0.527	0.465	0.469	0.539

The significance levels of 1%, 5%, and 10% are denoted by ***, **, and *, respectively. Standard errors clustered at the household level are reported in parentheses.

This study measures non-instrumental support from two dimensions: emotional support and perceived value support. Emotional support is assessed through three indicators. First, based on the response to “How would you describe your relationship with your father/mother (child) in the past 6 months?” a parent–child relationship index (*relation*) is constructed, ranging from 1 to 5, with higher values indicating closer relationships. Second, using the answer to “How often have you contacted your father/mother (child) via phone calls, text messages, letters, or emails in the past 6 months?,” an interaction frequency index (*connect*) is created. Third, based on “How often have you met your father/mother (child) in the past 6 months?,” a meeting frequency index (*meet*) is constructed. Both interaction and meeting frequency indices range from 1 to 7, and have been reverse-coded so that higher scores represent higher frequency. Columns (3)–(5) in Table 7 report the regression results of offspring employment stability on parental emotional support. The results show that the key explanatory variable-offspring employment stability-significantly and positively affects all three indicators, enhancing the parent–child relationship, daily communication frequency, and meeting frequency. This suggests that employment stability strengthens emotional bonds and interactive contact between parents and children, thereby reinforcing the emotional support system that positively influences parents’ physical and mental health.

Perceived value support reflects parents’ subjective evaluations of self-worth and life status and is measured across three aspects: perceived social status, life satisfaction, and happiness. Specifically, perceived social status (*status*) is derived from the question “How would you rate your social status in your local community?”; life satisfaction (*satisfy*) is based on “How satisfied are you with your life?” Both variables range from 1 to 5, with higher values indicating more positive subjective feelings. Happiness (*happy*) is measured by the question “How happy do you feel?” on a scale of 1–10, where higher values indicate greater happiness. Columns (6)–(8) in Table 7 present the regression results for perceived value support. The findings reveal that offspring employment stability significantly increases parents’ perceived social status, life satisfaction, and happiness. This indicates that employment stability enhances parents’ subjective sense of value, thereby improving their overall health status, and serves as a key mechanism through which offspring employment stability promotes intergenerational health transmission.

### 6.2 Heterogeneity analysis

#### 6.2.1 Gender heterogeneity in the effect of children’s employment stability on parental health

The previous empirical results demonstrate that offspring employment stability has a significant improvement effect on both the physical and mental health of parents. However, within the context of traditional Chinese family structures and the cultural norm of “raising sons to secure old age,” sons are typically assigned greater responsibility for parental support. This may lead to gender-based differences in how offspring employment stability influences parental health outcomes (48). With rising female labor force participation and the growth of flexible employment, daughters are more likely to engage in locally based or flexible jobs, allowing them to provide parental support through emotional interaction and daily care. In contrast, sons are more often engaged in full-time or migratory employment, constrained by time and distance, and thus primarily fulfill intergenerational responsibilities through financial means. Building on the theoretical analysis presented earlier, this paper further examines the marginal effects of sons’ and daughters’ employment stability on improving parental physical and mental health.

Table 8 presents the regression analysis based on Equations (3) and (4), reporting the differential effects of sons’ and daughters’ employment stability on parental physical and mental health. Column (1) presents the regression results for parental physical health, showing that compared to offspring with unstable employment, daughters’ employment stability significantly reduces parents’ hospitalization probability by an average of 19.9%. Among offspring with stable employment, sons’ employment stability further reduces parents’ hospitalization probability by 27.1%, which is 7.2 percentage points higher than that of daughters. These findings indicate that employment stability improves parental physical health regardless of offspring gender; however, the marginal health benefits are more pronounced for sons. Column (2) reports the effects on parental mental health. The results show that compared to offspring with unstable employment, daughters’ employment stability significantly lowers parents’ depression scores by 0.123 standard deviations. In contrast, the mental health improvement associated with sons’ employment stability is significantly weaker, reducing parents’ depression scores by approximately 0.033 standard deviations, a difference of about 0.09 standard deviations. These findings suggest that while offspring employment stability overall alleviates parental depressive symptoms, daughters exhibit a stronger marginal effect in improving parental mental health. The above conclusions partially validate hypotheses H_1_ and H_2_ proposed in this paper.

**Table 8:** Gender heterogeneity in the effect of children’s employment stability on parental health.

Variables	*old_health*	*old_cesd*
	(1)	(2)
*ch_stability*	−0.199***	−0.123***
	(0.020)	(0.030)
ch_stability*ch_man	−0.072***	0.090**
	(0.023)	(0.038)
Control	Yes	Yes
Individual FE	Yes	Yes
Year FE	Yes	Yes
*N*	20,993	20,993
Adj. *R*^2^	0.458	0.468

The significance levels of 1%, 5%, and 10% are denoted by ***, **, and *, respectively. Standard errors clustered at the household level are reported in parentheses. The regressions controlled for *ch_gender* in addition to the baseline control variables.

#### 6.2.2 Heterogeneity analysis of mechanisms

Based on the conclusions in Table 8 and the previous theoretical analysis of the structural division of intergenerational support, offspring gender may play a role in this division of labor. Sons primarily exert a more significant improvement effect on parental physical health through instrumental support such as economic assistance, whereas daughters tend to provide emotional companionship and psychological comfort-forms of non-instrumental support-demonstrating stronger effects on improving parental mental health (Li, 2021). Therefore, this section further explores the gender-differentiated pathways through which offspring employment stability influences parental health, deepening the understanding of how offspring employment stability improves parental health and revealing the gendered logic underlying intra-family resource allocation and caregiving responsibilities.

Table 9 presents the differential effects of offspring employment stability on parental physical and mental health between sons and daughters. Columns (1) and (2) show that sons’ employment stability has a more pronounced impact on enhancing both the likelihood and intensity of economic support to parents. Compared to daughters, sons with stable employment are 9.9% more likely to provide financial assistance and offer an average monthly economic support amount that is 19.9% higher. Columns (3)–(5) indicate that, relative to sons, daughters’ employment stability significantly improves the parent–child relationship, increases the frequency of daily interactions, and raises the frequency of face-to-face meetings. This suggests that daughters play a vital role in non-instrumental intergenerational support, helping to alleviate parents’ emotional stress and improve their mental health. These findings confirm the existence of a gender-based structural division of labor in family intergenerational support, supporting hypotheses H_1_ and H_2_. Columns (6)–(8) report that offspring employment stability significantly enhances parents’ perceived social status, life satisfaction, and happiness, indicating that stable employment promotes parents’ positive subjective evaluations and psychological well-being. However, no significant gender differences are observed in these subjective value perception supports, suggesting that both sons’ and daughters’ employment stability similarly contribute to improving parents’ subjective sense of value, consistent with hypothesis H_3_.

**Table 9:** Heterogeneity analysis of mechanisms.

Variables	Economic support		Emotional support			Perceived value support		
	*support1* (1)	*support2* (2)	*relation* (3)	*connect* (4)	*meet* (5)	*status* (6)	*satisfy* (7)	*happy* (8)
*ch_stability*	0.134***	0.264***	1.010***	0.638***	0.910***	0.143***	0.144***	0.535***
	(0.034)	(0.086)	(0.089)	(0.126)	(0.146)	(0.054)	(0.052)	(0.101)
ch_stability*ch_man	0.099***	0.199**	−0.889***	−0.488***	−0.563***	−0.093	−0.078	−0.021
	(0.038)	(0.101)	(0.096)	(0.144)	(0.163)	(0.062)	(0.058)	(0.115)
Control	Yes	Yes	Yes	Yes	Yes	Yes	Yes	Yes
Individual FE	Yes	Yes	Yes	Yes	Yes	Yes	Yes	Yes
Year FE	Yes	Yes	Yes	Yes	Yes	Yes	Yes	Yes
*N*	20,993	15,873	20,592	20,993	20,952	20,993	20,993	20,993
Adj. *R*^2^	0.240	0.311	0.312	0.312	0.530	0.465	0.468	0.540

The significance levels of 1%, 5%, and 10% are denoted by ***, **, and *, respectively. Standard errors clustered at the household level are reported in parentheses. The regressions controlled for *ch_gender* in addition to the baseline control variables.

In summary, the gender-specific mechanisms underlying the differential impact of offspring employment stability on parental physical and mental health are as follows: sons’ employment stability plays a more significant role in improving parental physical health, primarily through enhanced economic support; daughters’ employment stability is more effective in improving parental mental health, largely reflecting non-instrumental support such as emotional communication. This finding reveals the multidimensional pathways of intergenerational family support shaped by gendered role divisions and offers important insights for understanding intra-family resource allocation mechanisms and improving older adult care support systems in the context of population aging.

#### 6.2.3 Work-hour heterogeneity in the effect of children’s employment stability on parental health

This paper concludes with an exploratory analysis. Working hours exert dual effects-an income effect and a substitution effect-on the relationship between offspring employment stability and parental health. On one hand, longer working hours typically correspond to higher labor income, thereby enhancing offspring’s capacity to provide economic support to their parents. This income effect enables children to offer sustained and higher-quality financial assistance, improving parents’ living and medical conditions, which positively impacts their health. On the other hand, increased working hours reduce discretionary time, limiting opportunities for accompanying and caring for parents. This substitution effect weakens emotional communication and caregiving, potentially harming parental health. Moreover, excessively long working hours are often associated with high stress levels; children under prolonged work pressure may transmit negative emotions to their parents, increasing parental anxiety and emotional instability, thus undermining the beneficial health effects associated with offspring employment stability.

Based on this, the differential impact of offspring employment stability on parental health across working hours depends on the combined effects of income and substitution. Using the statutory 44-h workweek as the cutoff, the sample is divided into a standard hours group and an overtime group to examine how the health transmission effects of offspring employment stability vary under different labor intensities. The detailed regression results are presented in Table 10. The findings show that within the standard working hours (weekly working time not exceeding 44 h), the income effect outweighs the substitution effect, and offspring employment stability significantly improves parental physical and mental health. However, when offspring work overtime (weekly working time exceeding 44 h), the substitution effect dominates the income effect, and the positive impact of employment stability on parental health becomes insignificant, indicating that overtime work weakens the intergenerational health returns of stable employment. Therefore, while emphasizing the positive role of employment stability in promoting older population health, attention should also be paid to the moderating role of working hours on these health effects. Neglecting reasonable work hour arrangements may substantially diminish the intergenerational health benefits brought by stable employment due to the substitution effect caused by overtime work, ultimately affecting the sustainability and effectiveness of intergenerational support.

**Table 10:** Work-hour heterogeneity in the effect of children’s employment stability on parental health.

Variables	*old_health*		*old_cesd*	
	worktime>44 (1)	worktime<=44 (2)	worktime>44 (3)	worktime<=44 (4)
*ch_stability*	0.100	−0.253***	0.077	−0.105**
	(0.066)	(0.032)	(0.106)	(0.042)
Control	Yes	Yes	Yes	Yes
Individual FE	Yes	Yes	Yes	Yes
Year FE	Yes	Yes	Yes	Yes
*N*	6,859	12,714	3,859	12,714
Adj. *R*^2^	0.583	0.388	0.528	0.432

The significance levels of 1%, 5%, and 10% are denoted by ***, **, and *, respectively. Standard errors clustered at the household level are reported in parentheses.

## 7 Conclusion

Although both developed and developing countries are grappling with the challenges of population aging and its implications for public health, existing research has predominantly focused on developed economies such as the United States, Japan, and several European countries, often examining how adult children’s socioeconomic status influences parental health. In contrast, empirical evidence from China remains limited, particularly regarding the role of offspring employment stability in shaping the physical and mental well-being of older parents. Furthermore, the underlying mechanisms-especially the interplay of economic, emotional, and perceived value support-have not been fully explored. To address these gaps, this study draws on nationally representative data from the China Family Panel Studies (CFPS) to investigate both the direct effects and the heterogeneity of offspring employment stability on parental health, as well as the pathways through which these effects are transmitted.

This study utilizes data from the China Family Panel Studies (CFPS, 2016–2022) to examine how offspring employment stability affects parents’ physical and mental health and to uncover its underlying mechanisms. The results show that stable employment among adult children significantly improves parental health—reducing hospitalization probability by 24.9% and lowering depression scores by 0.063 standard deviations. These findings remain robust after multiple sensitivity tests and controlling for endogeneity. Further analysis reveals that employment stability promotes parental well-being mainly through three channels: enhanced economic, emotional, and perceived value support from children. Gender heterogeneity analysis indicates that sons primarily improve parents’ physical health through economic support, while daughters have a stronger positive impact on mental health via emotional support, with no significant gender difference in perceived value support. Additionally, working hours moderate these effects through competing “income” and “substitution” mechanisms—standard working hours reinforce the health benefits of employment stability, whereas overtime work weakens them.

Moreover, we note that although children’s employment stability significantly improves parental health through economic, emotional, and value-based support, some limitations remain. Health indicators in the CFPS are self-reported and may involve measurement error; unobserved regional policy differences could introduce potential endogeneity; and the limited observation period restricts long-term and spatial analysis. Future studies may incorporate objective health data, quasi-natural experiments, or cross-country comparisons to address these issues. Beyond China, our findings also have broader implications: improving job stability can enhance older population well-being through sustained support in family-oriented societies, while in welfare-based systems, ensuring job stability and work–life balance may serve as important public health strategies in aging societies.

These findings carry important policy implications. Policymakers should strengthen the family support system by acknowledging the gender-differentiated pathways through which children’s employment stability enhances parental health. Sons tend to contribute mainly through economic support, whereas daughters exert a stronger influence through emotional interaction and companionship. To foster a more balanced intergenerational support structure, targeted measures such as moderate reductions in medical expenses or tax incentives for parents of steadily employed children, along with the development of community-based or digital emotional companionship platforms to facilitate daughters’ involvement, should be encouraged. Moreover, since excessive overtime diminishes the positive spillovers of stable employment on parental well-being, stricter enforcement of working-hour regulations and effective sanctions for noncompliant enterprises are necessary. Promoting flexible work arrangements and family-friendly organizational practices can help employees better balance professional and caregiving responsibilities, thereby amplifying the intergenerational health benefits of employment stability within an aging society.

## Data Availability

The data analyzed in this study is subject to the following licenses/restrictions: the data used in this study are derived from the China Family Panel Studies (CFPS). Detailed information is available at https://cfpsdata.pku.edu.cn/#/home. Requests to access these datasets should be directed to https://cfpsdata.pku.edu.cn/#/home.

## Footnotes

1 WHO: number of people over 60 years set to double by 2050; major societal changes required. https://www.who.int/news/item/30-09-2015-who-number-of-people-over-60-years-set-to-double-by-2050-major-societal-changes-required?utm_source=chatgpt.com

2 China raises basic pension benefits for retirees. https://english.www.gov.cn/news/202507/10/content_WS686fa196c6d0868f4e8f40d4.html?utm_source=chatgpt.com

3 The China Family Panel Studies (CFPS) conducts a full-sample follow-up survey every 2 years; therefore, the data used in this study comprise pooled cross-sectional data from the 2016, 2018, 2020, and 2022 survey waves.

4 The method for constructing this indicator is as follows: (1) signing a labor contract (1 = yes, 0 = no), (2) job mobility (1 = no job change, 0 = job change), (3) income level (1 = above the sample median, 0 = below), and (4) social insurance participation (1 = yes, 0 = no). The total score ranges from 0 to 4, with higher values indicating greater employment stability.
